# Time Required for Extubation While Using Bispectral Index Monitoring Compared to End-Tidal Anesthetic Gas Concentration in Patients Undergoing General Anesthesia

**DOI:** 10.7759/cureus.86742

**Published:** 2025-06-25

**Authors:** Amithkumar SK, Deepak V Kadlimatti, Santosh Kumar, Palla Sree Divya, Mithra Ajith

**Affiliations:** 1 Department of Anaesthesiology, Dr. B.R. Ambedkar Medical College & Hospital, Bengaluru, IND

**Keywords:** bispectral index, end-tidal anesthetic, extubation time, gas concentration, isoflurane

## Abstract

Objective: The objective of this study was to compare extubation timings between patients monitored with the bispectral index (BIS) and those monitored using end-tidal anesthetic gas (ETAG) to determine which method provides a more efficient recovery profile.

Methodology: A prospective observational study was conducted on 50 patients aged 18 to 60 years, classified as American Society of Anesthesiologists (ASA) physical status I-II, undergoing elective surgeries under general anesthesia (GA). Patients were randomized into BIS and non-BIS (ETAG) groups to assess anesthetic consumption and recovery characteristics. Standard anesthetic protocols were followed. In the BIS group, BIS values were maintained between 40 and 60, while in the ETAG group, end-tidal isoflurane concentrations were kept between 0.7 and 1.3 minimum alveolar concentration (MAC). Intraoperative parameters and extubation times were recorded. Isoflurane administration was discontinued at the time of skin closure, and neuromuscular blockade was reversed once a train-of-four (TOF) ratio greater than 0.9 was confirmed. Data were analyzed using SPSS version 26.0 (IBM Corp., Armonk, NY), with a p-value less than 0.05 considered statistically significant.

Results: There was no statistically significant difference in the overall duration of anesthesia between the two groups (p > 0.05). However, the BIS group had a significantly shorter extubation time, averaging 148.4 ± 30.5 seconds, compared to 201.2 ± 43.6 seconds in the ETAG group. The mean difference was 52.8 ± 13.1 seconds (p = 0.001). Additionally, the average hourly isoflurane consumption was significantly lower in the BIS group (5.9 ± 0.77 mL) compared to the ETAG group (6.56 ± 1.12 mL), with a mean difference of 0.66 ± 1.04 mL (p = 0.01).

Conclusion: Extubation occurred significantly earlier in patients monitored with BIS than in those observed with ETAG. Furthermore, BIS-guided anesthesia was associated with reduced isoflurane consumption compared to ETAG-guided monitoring.

## Introduction

Rapid and smooth recovery following general anesthesia is a desirable goal associated with improved clinical outcomes and more efficient use of hospital resources [1]. Early recovery facilitates better utilization of operating rooms, shorter stays in the intensive care unit (ICU) and hospital, and overall cost-effectiveness [2,3]. Achieving this requires a careful balance between maintaining adequate anesthetic depth during surgery and minimizing excessive drug administration that may delay emergence [4].

Traditionally, anesthesiologists have relied on clinical signs such as heart rate, blood pressure, patient movement, and secretions to assess anesthetic depth [5]. However, these indicators are subjective and may be influenced by non-anesthetic factors such as hypovolemia, bladder distension, or pain, rendering them unreliable [5]. Various monitoring devices have been developed to assess anesthetic depth and guide drug titration to improve accuracy [2,3]. Among these, bispectral index (BIS) and end-tidal anesthetic gas (ETAG) concentration monitoring are widely used [3].

BIS is an electroencephalogram (EEG)-based monitor that analyzes frontal EEG signals to generate a numerical value between 100 and 0, where 100 indicates full consciousness and 0 represents complete cortical inactivity [6,7]. A BIS range of 40 to 60 is optimal for general anesthesia, helping to prevent intraoperative awareness while minimizing anesthetic drug use [6,7]. BIS integrates multiple EEG parameters and correlates well with sedation levels and responsiveness [4]. It is also relatively insensitive to the specific anesthetic agent used. By enabling individualized titration of inhaled anesthetics, BIS has been associated with reduced anesthetic consumption and shorter recovery times [8,9].

ETAG monitoring, by contrast, provides an indirect estimate of the brain’s anesthetic concentration by measuring anesthetic gas levels in exhaled air using infrared gas analyzers [10]. The most commonly used metric in this method is the minimum alveolar concentration (MAC), and maintaining ETAG between 0.7 and 1.3 MAC is thought to minimize the risk of intraoperative awareness while ensuring adequate anesthesia [6,7]. ETAG monitoring is widely accepted as a practical and effective approach for assessing anesthetic depth during inhalational anesthesia.

Although both BIS and ETAG guide anesthetic delivery, limited evidence compares their effectiveness in promoting early recovery [10]. Time to extubation and overall recovery are influenced by various factors, including patient characteristics, duration of surgery, and anesthetic techniques and agents used. Among inhalational agents, desflurane is often favored for early recovery due to its low blood-gas partition coefficient, which allows for rapid elimination following discontinuation [11].

Existing literature has predominantly evaluated these modalities separately or with agents like desflurane, which is known for faster elimination. However, isoflurane remains widely used in many clinical settings [4,10], and direct comparisons of BIS- and ETAG-guided anesthesia using isoflurane are limited. This highlights the novelty of evaluating extubation times and recovery profiles between the two monitoring strategies in this specific context.

This study aimed to compare the time required for extubation in patients undergoing elective surgeries under isoflurane-based general anesthesia when guided by BIS monitoring versus ETAG concentration.

## Materials and methods

Study design

This prospective observational study was conducted at a tertiary care center after obtaining approval from the Institutional Ethics Committee of Dr. B.R. Ambedkar Medical College & Hospital (Approval No.: EC 454). The study was registered with the Clinical Trials Registry of India (CTRI/2024/04/065523).

Inclusion and exclusion criteria

Patients aged 18 to 60 years of either gender, classified as American Society of Anesthesiologists Physical Status (ASA-PS) I or II, and scheduled for elective procedures under general anesthesia were included. Exclusion criteria included a history of prolonged use of anticonvulsants, opioids, benzodiazepines, or alcohol; pre-existing hepatic, renal, or cardiac conditions; and cognitive or neurological impairments, including dementia or stroke-related abnormalities.

Sample size calculation

The sample size was calculated based on a type I error (α) of 0.05 and a type II error (β) of 0.10, which provides 80% power. Assuming a standard deviation (SD) of 2 and a minimum detectable difference (d) of 2.1, the formula used was:

\[n = \frac{2 \times (Z_{\alpha/2} + Z_{\beta})^2 \times SD^2}{d^2}\]

Substituting the values:

\[ n = \frac{2 \times (1.96 + 1.28)^2 \times 4}{4.41} \approx 21 \text{ patients per group}\]

To allow for potential dropouts, 25 patients were recruited in each group, making a total sample size of 50.

Preoperative protocol and randomization

**Figure 1:**
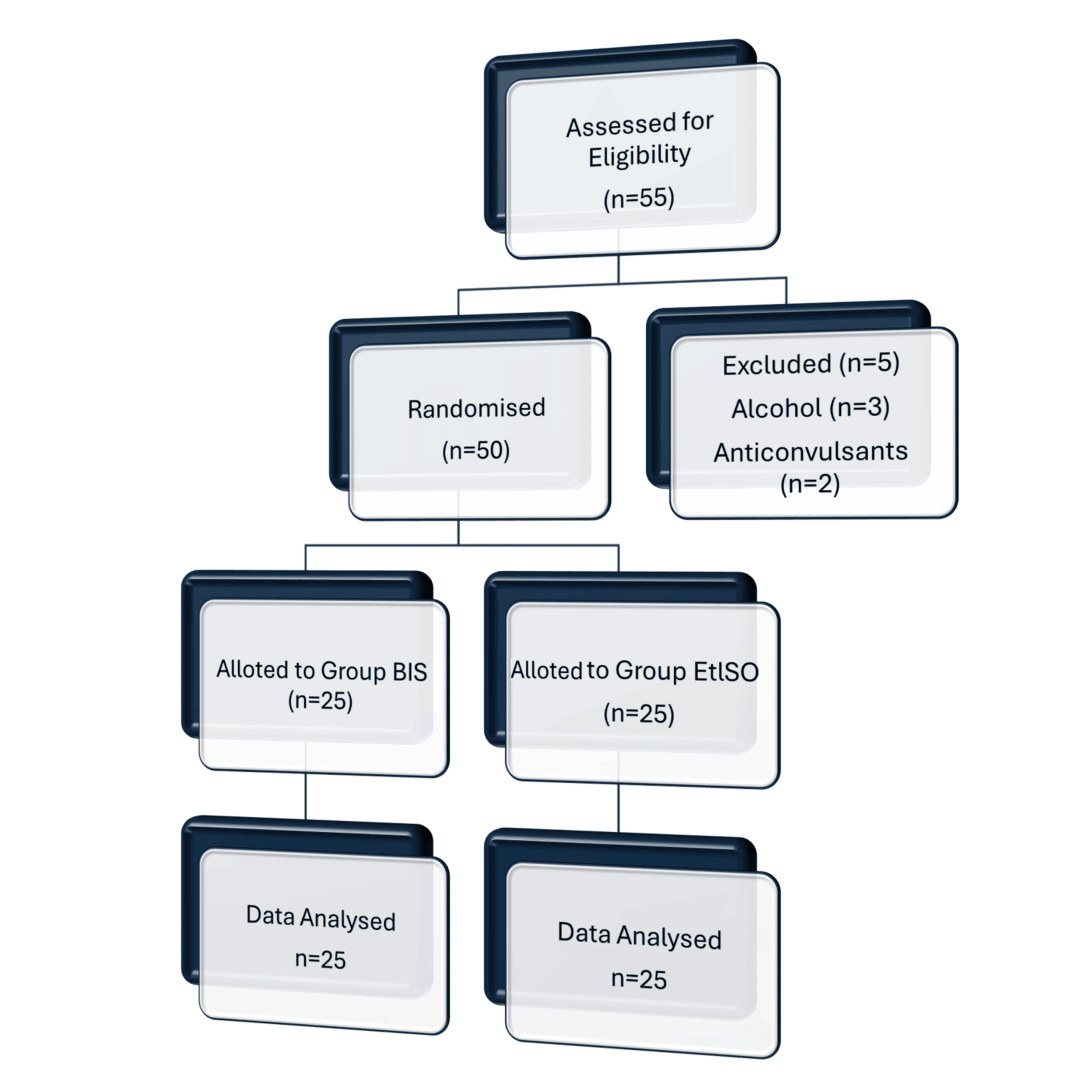
CONSORT flowchart. CONSORT: Consolidated Standards of Reporting Trials; BIS: bispectral index; ETISO: end-tidal isoflurane.

All patients received 0.25 mg of oral alprazolam the night before surgery. Before the induction of anesthesia, a minimum fasting duration of six hours was required. Patients were randomly assigned to the BIS or non-BIS group using a computer-generated randomization method. Allocation concealment was maintained through sealed opaque envelopes to reduce selection bias. Although complete blinding was not possible due to the nature of BIS monitoring, the attending anesthesiologists and surgeons were not informed of the group allocation during the intraoperative and extubation period to minimize performance bias (Figure 1).

Anesthesia procedure

Standard monitoring was employed upon arrival in the operating room, including ECG, noninvasive blood pressure, and pulse oximetry. A BIS™ Quatro sensor (Medtronic, Dublin, Ireland) was applied to the left forehead of all patients; however, BIS readings were visible only in the BIS group. Preoxygenation was followed by induction with intravenous propofol (2 mg/kg), fentanyl (2 µg/kg), and succinylcholine (2 mg/kg). Endotracheal intubation was performed using appropriately sized tubes, and neuromuscular blockade was maintained with atracurium (0.5 mg/kg). Anesthesia was maintained using a mixture of oxygen, air, and isoflurane, with supplemental doses of atracurium as needed. Intraoperative parameters, including heart rate (HR), systolic blood pressure (SBP), diastolic blood pressure (DBP), mean arterial pressure (MAP), peripheral oxygen saturation (SpO₂), end-tidal carbon dioxide (ETCO₂), fresh gas flow (FGF), BIS values, minimum alveolar concentration (MAC), and neuromuscular transmission (NMT), were recorded at five-minute intervals.

In the BIS group, the depth of anesthesia was adjusted to maintain BIS values between 40 and 60. In the non-BIS group, BIS monitoring was not used, and isoflurane was titrated to maintain an age-adjusted end-tidal concentration of 0.7-1.3 MAC. The BIS monitor and anesthetic gas analyzer were calibrated prior to the start of each case, per manufacturer recommendations, to ensure the accuracy and consistency of readings across all patients.

Anesthetic agent consumption

Total and hourly isoflurane consumption was calculated for each patient using the integrated volatile agent consumption monitor on the anesthesia workstation (Primus®, Dräger, Lübeck, Germany/Datex-Ohmeda®, GE HealthCare, Chicago, IL). These systems compute agent use based on real-time data from the vaporizer setting, FGF, and administration time. Hourly consumption was calculated by dividing the total consumption (in mL) by the total anesthesia duration in hours.

Post-surgical data collection

Isoflurane was discontinued at the time of skin closure. The FGF was increased to 4 L/min with 100% oxygen. Tracheal extubation was performed once clinical criteria were met. The duration of surgery and anesthesia was recorded in minutes. The time to extubation was defined as the interval from discontinuing the inhalational agent to successful tracheal extubation. It was measured using a digital stopwatch and served as the primary indicator of recovery time. Extubation assessments were performed by an independent observer trained in airway management, and a second observer independently confirmed the findings to enhance inter-rater reliability.

Neuromuscular blockade was reversed with neostigmine (0.05 mg/kg) and glycopyrrolate (0.01 mg/kg) after confirming a train-of-four (TOF) ratio > 0.9 and allowing sufficient time, at least 30 minutes, since the last dose of muscle relaxant (atracurium). A three- to five-minute waiting period was routinely observed after achieving TOF > 0.9 to confirm the return of airway reflexes, adequate spontaneous ventilation, and overall clinical readiness prior to extubation. Each patient's total and hourly consumption of inhalational agents was determined. Consumption time was calculated using the agent-specific consumption data provided by the anesthesia workstation, which incorporates agent concentration, FGF rate, and duration of administration. Hourly consumption was derived by dividing total usage by the duration of anesthesia.

Extubation criteria

Extubation was carried out upon fulfillment of the following criteria: inspiratory pressure < -25 cmH₂O, tidal volume > 5 mL/kg, vital capacity > 10 mL/kg, rapid shallow breathing index (RSBI) < 100, and TOF ratio > 0.9.

Although a TOF ratio > 0.9 indicates adequate neuromuscular recovery, extubation was sometimes delayed beyond this threshold to ensure full clinical readiness, such as adequate consciousness, protective airway reflexes, and spontaneous respiratory effort, essential for safe extubation.

Statistical analysis

Statistical analysis was performed using SPSS version 26.0 (IBM Corp., Armonk, NY). A p-value < 0.05 was considered statistically significant. Continuous variables were compared between groups using the independent t-test, while categorical variables were analyzed using the chi-square test. The null hypothesis was that the two monitoring approaches would produce no significant difference in recovery times.

## Results

Baseline characteristics were comparable between the end-tidal isoflurane (ETISO) (N = 25) and BIS (N = 25) groups. Mean age and weight did not differ significantly (p = 0.21 and 0.31, respectively). Males were more common in the BIS group, with 17 (68%) compared to nine (36%) in the ETISO group. Hypothyroidism was observed in 10 (40%) patients in the ETISO group, whereas none were reported in the BIS group. Type 2 diabetes was present in four (16%) patients in the BIS and ETISO groups. Most patients had no comorbidities: 18 (72%) in the ETISO group and 20 (80%) in the BIS group. Smoking was reported in two (8%) ETISO patients and none in the BIS group. No statistically significant differences were noted between groups (all p > 0.05) (Table 1).

**Table 1:** Baseline demographic and clinical characteristics of patients in the ETISO and BIS groups. ETISO: end-tidal isoflurane; BIS: bispectral index. Data have been represented as N (%) and mean and standard deviation (mean ± SD).

Variables	ETISO (N = 25)	BIS (N = 25)	p-value
Age in years (mean ± SD)	41.04 ± 11.7	36.96 ± 10.6	0.21
Weight (mean ± SD)	66.8 ± 7.21	69.12 ± 8.6	0.31
Sex			0.76
Male	9 (36%)	17 (68%)	
Female	16 (64%)	8 (32%)	
Comorbidities			0.26
Hypertension	2 (8%)	1 (4%)	
Hypothyroidism	10 (40%)	0 (0%)	
Known case of type 2 diabetes	1 (4%)	3 (12%)	
Known case of thyroid	0 (0%)	1 (4%)	
Type 2 diabetes	3 (12%)	0 (0%)	
Nil	18 (72%)	20 (80%)	
Habits			0.14
Smoking	2 (8%)	0 (0%)	
Nil	23 (92%)	25 (100%)	

The majority of patients in both groups had a Mallampati grade 2 classification, with 21 patients (84%) in the BIS group and 22 patients (88%) in the ETISO group. Similarly, ASA-PS grade 1 was more common in both groups, observed in 21 (84%) BIS and 18 (72%) ETISO patients. No statistically significant differences were found between the groups in Mallampati or ASA-PS grading (p = 0.14 and p = 0.30, respectively) (Table 2).

**Table 2:** Comparison of MP grade and ASA-PS grading between the BIS and ETISO groups. ETISO: end-tidal isoflurane; BIS: bispectral index; MP grade: Mallampati grading; ASA-PS grading: American Society of Anesthesiologists physical status classification. Data have been represented as N (%). P < 0.05 was considered significant.

Grading	BIS	ETISO	p-value
MP grade			0.14
1	4 (16%)	3 (12%)	
2	21 (84%)	22 (88%)	
ASA-PS grading			0.30
1	21 (84%)	18 (72%)	
2	4 (16%)	7 (28%)	

The duration of surgery was slightly longer in the BIS group (96.8 ± 12.8 minutes) compared to the ETISO group (90.8 ± 12.8 minutes), with a mean difference of -6.0 minutes; however, this was not statistically significant (p = 0.10). Similarly, the total duration of volatile anesthesia was comparable between groups: 82.4 ± 11.0 minutes in the BIS group and 79.8 ± 11.9 minutes in the ETISO group (p = 0.42). No significant differences were observed for either parameter (Table 3).

**Table 3:** Comparison of the duration of surgery and time of volatile anesthesia between the ETISO and BIS groups. ETISO: end-tidal isoflurane; BIS: bispectral index. Data have been represented as mean and standard deviation (mean ± SD). P < 0.05 was considered significant.

Variables	ETISO group (Mean ± SD)	BIS group (Mean ± SD)	95% confidence interval	Difference	T-test value	p-value
Duration of surgery (min)	90.8 ± 12.8	96.8 ± 12.8	-13.2 to 1.29	-6.0	-1.65	0.10
Total time of volatile anesthesia	79.8 ± 11.9	82.4 ± 11.0	-9.13 to 3.93	-2.6	-0.8	0.42

Extubation time was significantly shorter in the BIS group (148.4 ± 30.5 minutes) compared to the ETISO group (201.2 ± 43.6 minutes), with a mean difference of 52.8 minutes (p = 0.001). The total inhalation agent volume consumed was lower in the BIS group at 7.76 ± 1.51 ml versus 8.65 ± 1.52 ml in the ETISO group (p = 0.04). Additionally, inhalation agent volume consumed per hour was significantly reduced in the BIS group (5.87 ± 0.76 ml/h) compared to the ETISO group (6.57 ± 1.12 ml/h; p = 0.01). All these differences were statistically significant (Table 4).

**Table 4:** Comparison of extubation time, inhalational agent volume consumed, and inhalational agent volume consumed per hour. ETISO: end-tidal isoflurane; BIS: bispectral index. Data have been represented as mean and standard deviation (mean ± SD). * shows a significant p-value. P < 0.05 was considered significant.

Variables	ETISO group (Mean ± SD)	BIS (Mean ± SD)	Difference	95% confidence interval	T-test value	p-value
Extubation time	201.2 ± 43.6	148.4 ± 30.5	52.8	31.39 to 74.2	4.96	0.001*
Inhalation agent volume consumed (ml)	8.65 ± 1.52	7.76 ± 1.51	-0.23	1.09 to 1.63	-0.52	0.04*
Inhalation agent volume consumed per hour	6.57 ± 1.12	5.87 ± 0.76	0.17	0.37 to 0.71	0.61	0.01*

## Discussion

The comparison of extubation and recovery durations between groups indicated that both BIS and ETAG monitoring yield comparable recovery results. Nonetheless, BIS-guided anesthesia consistently demonstrated benefits in minimizing recovery duration and anesthetic usage. The time until tracheal extubation was significantly shorter in the BIS-guided group compared to the ETISO group in the current study. This finding aligns with Shukla et al. [12], who reported a similar reduction in extubation duration with BIS monitoring. They concluded that BIS provides a more accurate assessment of anesthetic depth, enabling timely dosage adjustments and faster recovery. Our data showed that extubation occurred earlier in patients monitored with BIS than in those observed with ETAG under isoflurane-based general anesthesia. BIS values in the study groups ranged from 40 to 60, while the anesthetic concentration in the ETAG group was maintained between 0.7 and 1.3 MAC.

BIS measurements were obtained upon achieving steady-state concentrations of volatile anesthetics. At comparable MAC levels of halothane and isoflurane, BIS values were lower with isoflurane. Furthermore, at equi-MAC, sevoflurane produced lower BIS values during both wash-in and wash-out phases than isoflurane; however, both agents exhibited cardio stability. These pharmacodynamic properties emphasize the need for personalized titration guided by objective monitoring tools such as BIS.

Jain et al. [13] demonstrated that BIS-guided protocols in isoflurane anesthesia significantly reduced extubation time and overall anesthetic consumption compared to MAC-based methods. Their study highlighted that BIS monitoring facilitates more precise anesthetic administration, resulting in quicker patient recovery and reduced drug use. This study supports our observation that BIS improves anesthetic depth regulation, enhances recovery profiles, optimizes resource efficiency, and facilitates smoother perioperative outcomes. Our findings are corroborated by Gu et al. [14], who showed that BIS monitoring improves the accuracy of anesthetic depth management and enables faster emergence and extubation without compromising patient safety. These data reinforce that BIS allows more individualized and effective anesthetic titration than reliance on end-tidal gas concentrations alone.

A prior randomized trial reported significantly shorter extubation times in BIS-guided patients (17.5 minutes vs. 75 minutes; p < 0.001) despite no difference in total anesthetic doses between groups. No cases of intraoperative awareness were documented, indicating that BIS monitoring ensures patient safety while promoting faster recovery. Bagle et al. [15] also noted that BIS-guided isoflurane anesthesia significantly accelerated recovery and extubation times compared to MAC monitoring, attributing this to enhanced control of anesthetic depth and reduced anesthesia exposure duration.

However, some studies have reported different findings. Chaudhuri et al. [16] compared BIS and MAC monitoring during inhalational anesthesia and found no statistically significant difference in extubation time, though BIS showed a trend toward faster recovery [12]. These discrepancies might be explained by differences in study design, patient characteristics, anesthetic agents, or intraoperative management protocols. Persec et al. [17] also supported our results by showing reduced extubation times and decreased analgesic requirements in BIS-monitored patients, underscoring its role in improving overall perioperative recovery.

Particular attention is needed for geriatric patients, who often have reduced physiological reserves and comorbidities requiring careful anesthetic management. BIS monitoring in older Asian cohorts has been shown to minimize isoflurane use by up to 40%, resulting in faster awakening and smoother recovery profiles. This highlights the potential of BIS to reduce anesthetic exposure and side effects in vulnerable populations.

Although BIS monitoring is often emphasized for its superiority in tailoring anesthetic depth, ETAG-guided anesthesia has also proven effective in facilitating early postoperative recovery. In our study, hypothyroidism was present in 40% of patients in the ETISO group, while none were reported in the BIS group. Although all affected patients were biochemically euthyroid and under stable medical management preoperatively, this imbalance could have influenced anesthetic metabolism and recovery profiles, thereby acting as a potential confounder. Studies have indicated that end-tidal anesthetic concentration-guided anesthesia is comparable to BIS-guided protocols in patients receiving nitrous oxide and desflurane [2,9,12,18]. This result suggests ETAG may be a viable alternative when BIS is unavailable or cost-prohibitive.

Limitations

This study has several limitations. First, excluding patients with significant comorbidities such as hepatic or renal impairment, while methodologically intended to reduce confounding, may limit the generalizability of the findings to broader, real-world populations. Second, the sample size was relatively small and derived from a single tertiary care center, which may further restrict external validity. Additionally, an unequal distribution of sex between the groups (68% male in the BIS group vs. 36% in the ETISO group) and the presence of hypothyroidism in 40% of patients in the ETISO group, absent in the BIS group, could have introduced confounding effects on anesthetic metabolism and recovery profiles. Although these patients were clinically euthyroid, the imbalance remains a potential source of bias. The study also focused exclusively on elective surgeries performed under isoflurane-based anesthesia, so the findings may not apply to other agents like desflurane or sevoflurane, which have different pharmacokinetic and pharmacodynamic properties, or to emergency procedures involving different physiological stressors. Furthermore, the study did not evaluate long-term postoperative cognitive function, patient satisfaction, or the potential economic implications of earlier recovery. Future multicenter trials with larger, more diverse populations and standardized anesthetic protocols are warranted to validate and extend these results.

## Conclusions

This study demonstrated that BIS-guided anesthesia is associated with significantly faster extubation times and reduced isoflurane consumption compared to ETAG-guided monitoring in patients undergoing elective surgeries under general anesthesia. Although the overall duration of surgery and anesthesia was similar between the two groups, the BIS group achieved earlier extubation by an average of 52.8 seconds and consumed less total and hourly inhalational agent volumes. These findings suggest that BIS monitoring offers advantages in optimizing anesthetic delivery and enhancing perioperative recovery efficiency. However, the clinical applicability of these findings should be interpreted in light of the study’s limitations, including small sample size and group imbalances. Larger, multicenter studies are recommended to confirm these results and support the integration of BIS monitoring into routine anesthetic practice where appropriate.
